# Case Report: Afatinib Sensitivity in Rare EGFR E746_L747delinsIP Mutated LUAD With Peritoneal Metastases

**DOI:** 10.3389/fonc.2022.861271

**Published:** 2022-05-31

**Authors:** Lili Zhang, Lu Yang, Binxu Sun, Yixiao Deng, Jie Yang, Dongfang Wu, Fanming Kong

**Affiliations:** ^1^ Department of Oncology, First Teaching Hospital of Tianjin University of Traditional Chinese Medicine, Tianjin, China; ^2^ National Clinical Research Center for Chinese Medicine Acupuncture and Moxibustion, Tianjin, China; ^3^ The Genetic Analysis Department, YuceBio Technology Co., Ltd., Shenzhen, China

**Keywords:** lung adenocarcinoma, peritoneal carcinomatosis, sensitive, afatinib, EGFR E746_L747delinsIP

## Abstract

Patients with non-small cell lung cancer harboring the epidermal growth factor receptor (EGFR)-sensitive mutations are known to benefit significantly from EGFR tyrosine kinase inhibitors (TKIs), such as erlotinib, gefitinib, icotinib, or afatinib. However, the efficacy of EGFR-TKIs against rare mutations has not yet been well investigated. Here, we report a female patient with advanced lung adenocarcinoma (LUAD), carrying a rare mutation of *EGFR* Exon19 E746_L747delinsIP, who was administered first-generation EGFR-TKIs as the first-line treatment. The patient continued to progress slowly until peritoneal metastases have occurred. Subsequently, the patient was treated with anlotinib for 5 months until disease progression. Given the finding of the same EGFR rare mutation in peritoneal effusion without other EGFR-TKI resistance mutations, the patient received afatinib with a tremendous response. Our results may be of clinical relevance for patients with LUAD carrying this rare mutation, and these findings warrant further investigation.

## Introduction

Epidermal growth factor receptor (EGFR) tyrosine kinase inhibitor (TKI) targeting therapy has shown promise in the treatment of non-small cell lung cancer (NSCLC) with common *EGFR* mutations, such as L858R and exon 19 deletion (exon 19del) (1). With the rapid development of next-generation sequencing (NGS) technology, many atypical mutations have been identified in *EGFR* exons 18, 19, 20, and 21, respectively, but their sensitivity to TKIs is unclear. In the current study, afatinib, in particular, has demonstrated clinical efficacy in the treatment of some uncommon mutations, compound mutations, and some exon 20 insertions (2, 3).

Previous studies have shown that NSCLC with peritoneal metastases is rare (approximately 2%) (4) and has a poor prognosis with a median overall survival (mOS) of less than 3 months (5). Peritoneal metastases from NSCLC are often complicated by peritoneal effusion. Once malignant ascites occurs, it usually means that patients are in poor physical condition and lose the opportunity for treatment (6). Owing to the lack of experience in treating peritoneal complications, oncologists face a great challenge in the treatment of NSCLC with peritoneal metastases (7). Here, we report the case of a patient carrying *EGFR* E746_L747delinsIP mutated lung adenocarcinoma (LUAD) with peritoneal metastases that was successfully treated with the second-generation EGFR TKI targeted therapy, afatinib.

## Case presentation

**Figure 1:**
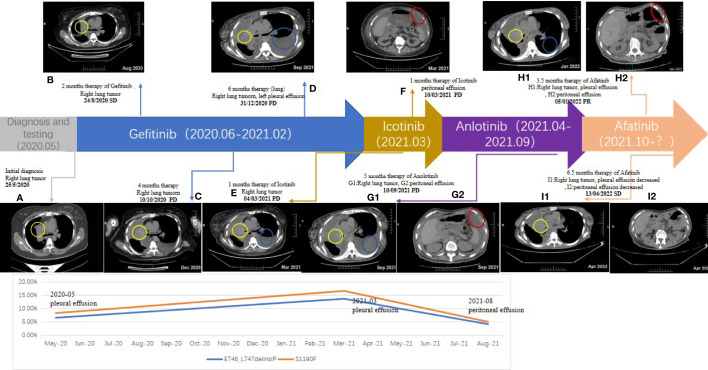
Timeline of the patient: Chest and Abdominal contrast-enhanced CT **(A–F, G1, G2, H1, H2, I1, I2)**; yellow circle: right lung tumor; blue circle: left pleural effusion; red circle: peritoneal effusion.

A 57-year-old Chinese woman without a smoking history or family history of cancer suffered from a cough in January 2020. In April 2020, computed tomography (CT) scans revealed right atelectasis, lung neoplasms, and pleural effusion (**Figure 1A**). Whole-body bone scans and magnetic resonance imaging of the brain revealed no evidence of metastasis. Using immunohistochemistry, the patient was diagnosed with LUAD (stage IV). The E746_L747delinsIP (6.6%) at exon 19 and the S1190F (1.74%) at exon 28 of the *EGFR* were identified in the pleural effusion biopsy specimen by NGS sequencing with a panel covering 525 cancer-related genes. None of the mutations were associated with EGFR-TKI primary resistance. Based on this result, the patient started treatment with gefitinib (250 mg/day) in June 2020; however, no radiological response was observed during treatment. Unfortunately, she experienced slow progressive disease (PD) with increasing lung neoplasms and emerging left pleural effusion in December 2020 (**Figures 1B–D**). The patient was then switched to icotinib in February 2021, because *EGFR* E746_L747delinsIP was misunderstood to be an *EGFR* exon 19 del, which is sensitive to the first-generation of EGFR-TKIs. However, PD was confirmed by CT showing peritoneal metastases in March 2021 (**Figures 1E, F**).

**Figure 2:**
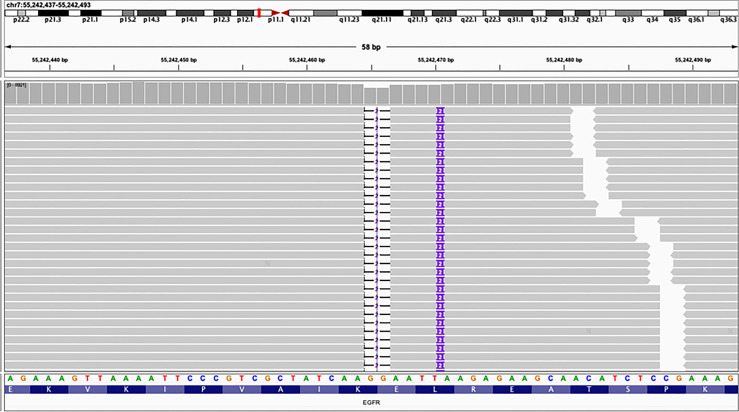
Next-generation sequencing (NGS) panel result showed an epidermal growth factor receptor (EGFR) mutation E746_L747delinsIP *via* simultaneous deletion and insertion of DNA fragments of 2 bp in exon 19.

**Figure 3:**
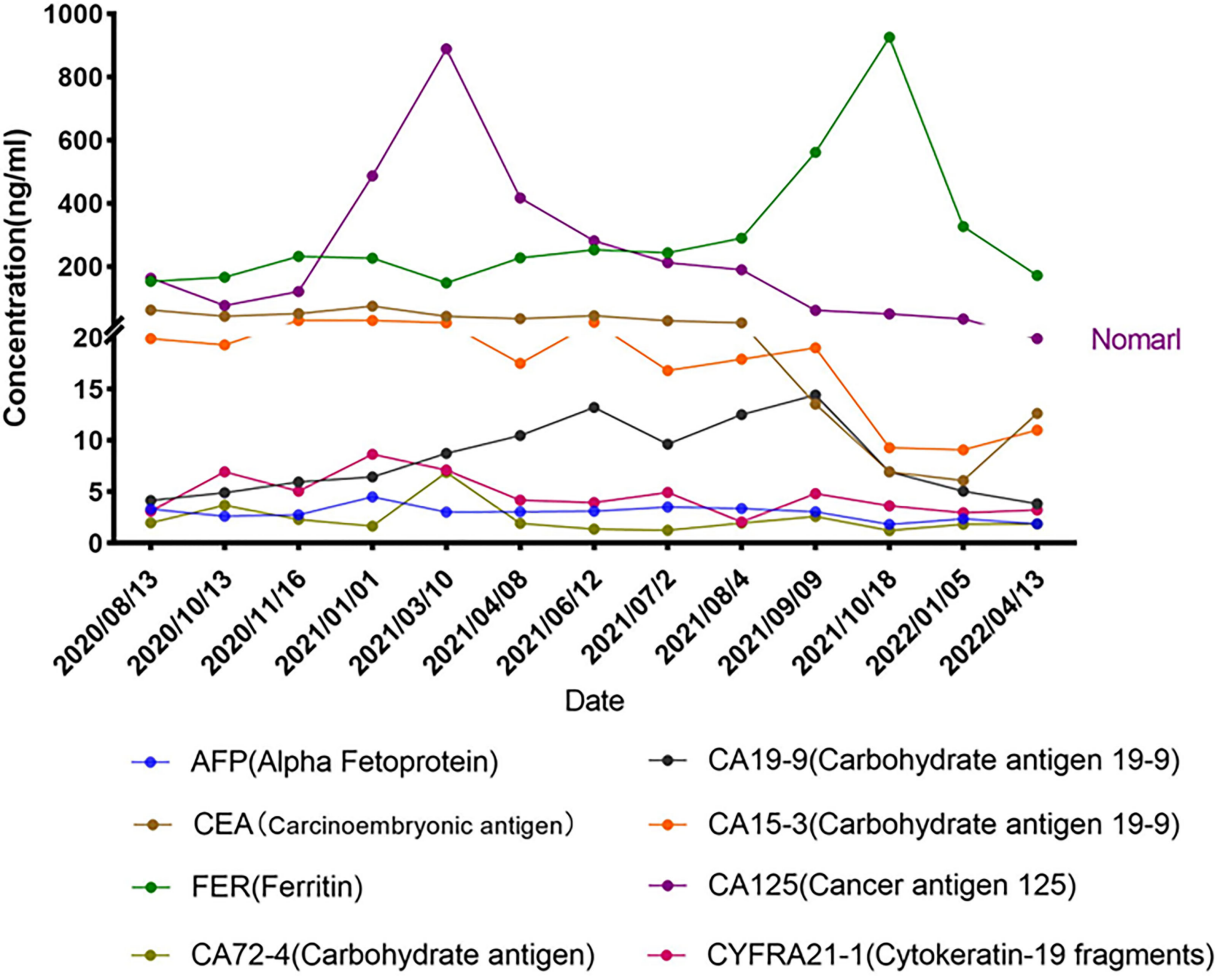
Serum tumor biomarkers during treatment.

Repetitive NGS analysis using pleural effusion detected *EGFR* E746_L747delinsIP (13.7%) in exon 19 and S1190F (2.95%) in exon 28 with increasing variant allele frequencies (VAF) as per a small, customized panel covering 74 cancer target related genes in Yucebio Technology (**Figure 2**). A timeline illustrating the patient’s medical history and therapy is presented in **Figure 1**. The patient was currently exhibiting PD. Some studies have suggested that anlotinib may provide survival benefits to patients with NSCLC with abdominal or pleural effusion (6, 8). Considering that the patient may not tolerate the adverse effects of afatinib, as well as refractory ascites, pleural effusion, and an Eastern Cooperative Oncology Group (ECOG) Performance Status score of 3, she received anlotinib (12 mg/day) in April 2021. During the treatment, the patient’s chest symptoms were relieved, and simultaneously, CA125 and CYFRA21-1 showed a decreasing trend, especially CA125 (**Figure 3**); however, the peritoneal effusion with a chylous appearance continued to increase (**Figure 1G2**), requiring drainage of 1000–1500 ml per day. Anlotinib treatment was discontinued in September 2021 because of PD.

Peritoneal effusions were collected for further genetic analysis. As shown in **Figure 1**, *EGFR* E746_L747delinsIP (4.2%) and S1190F (0.88%) were found again without other mutations, indicating that the LUAD metastasized to the peritoneum. Afatinib (40 mg/day) was administered in September 2021. The patient had received afatinib for approximately 3.5 months and achieved a partial response with a significant improvement in ascites and right pleural effusion. The ascites gradually diminished, and only a small amount of ascites was observed within the 1.5 months before the final test (**Figures 1H1, H2**). Serum tumor biomarkers showed a downward trend, especially CA125, decreasing to the average level for the first time (**Figure 3**). The treatment rapidly improved her clinical symptoms, including abdominal distension and appetite, despite the diarrhea. Her general condition improved from 3 to 2 according to the Eastern Cooperative Oncology Group Performance Status score. Since November 2021, the patient has had no further drainage of her ascites or pleural effusion, and the effusion showed a decreasing tendency. Follow-up CT until April 2022 showed that the pulmonary lesions were slightly enlarged, bilateral pulmonary nodules were increased, abdominal nodules continued to be stable, and the curative effect assessment of lesions primarily showed an increasing trend within the stable disease (SD) range (**Figures 1I1, I2**). Furthermore, the serum tumor biomarkers CEA and CYFRA21-1 showed an increase (**Figure 3**). In accordance with the patient’s and her family’s wishes, we performed a gene test using NGS again to explore if the patient developed drug-resistant mutations.

## Discussion

With the increasing popularity of NGS, uncommon *EGFR* mutations have been detected. However, some rare mutations lack clinical efficacy data, leading to the demand for continuous clinical reporting. Our case may be able to illustrate the sensitivity of the rare EGFR E746_L747delinsIP mutation to the efficacy of the first- and the second-generation EGFR-TKIs.

Here, we report the first efficacy data of an NSCLC patient carrying *EGFR* E746_L747delinsIP using the NGS platform. This mutation induces a double amino acid substitution (E746I-L747P) *via* the simultaneous deletion and insertion of two nucleotides at different positions in *EGFR* exon 19 (**Figure 2**). According to the Human Genome Variation Society (HGVS), this mutation is named *EGFR* E746_L747delinsIP (9). We also found a co-mutation *EGFR* S1190F, an uncertain clinically significant mutation, without any functional studies to affect the kinase domain of the EGFR or the efficacy of EGFR-TKIs.

In our case, the patient received gefitinib but continued to progress slowly, and according to the NCCN, it is a viable strategy for limited metastatic NSCLC to continue taking the first-generation EGFR-TKI. Therefore, icotinib was administered. A high frequency of *EGFR* E746_L747delinsIP mutation was found in ascites without other known TKI resistance mutations, indicating that *EGFR* E746_L747delinsIP might be resistant to first-generation TKIs. Pleural and ascites metastases from LUAD are relatively rare in patients with NSCLC. It has an inferior prognosis, with a mOS of approximately 1.3 months in the best supportive care group (7). We detected the same rare EGFR mutations in the patient’s pleural fluid and ascites samples. It is plausible that *EGFR* E746_L747delinsIP is sensitive to afatinib owing to its remarkable polyserous effusions and abdominal response.

To date, no actual efficacy data have been reported for patients with NSCLC with *EGFR* E746_L747delinsIP. Koopman et al. (2021) considered it to be comparable to L747P and predicted it to be sensitive to EGFR TKI, and mentioned that *EGFR* E746_L747delinsIP was an uncommon, actionable mutation (10). Additionally, there are still some clinical case reports on L747P and found that the current assessment of L747P efficacy for the first- and third-generation EGFR-TKIs is inconsistent (11–13). Moreover, Coco et al. (2015) reported a similar case in which a patient carrying an *EGFR* E746V-L747P (E746_L747delinsVP) activating mutation caused by four continuous nucleotides (AATT > TTCC) was resistant to gefitinib. He predicted that *EGFR* E746V-L747P was resistant to gefitinib *via* structure prediction (14). The phenomenon presented in our case was similar to that described above. Therefore, we speculate that *EGFR* E746_L747delinsIP is a primary resistance variant for the first-generation EGFR-TKIs.

In addition, most case reports suggest that *EGFR* L747P is sensitive to afatinib *via* the Uncommon *EGFR* Mutations Database. Retrospective studies suggest that afatinib has clinical activity in NSCLC with uncommon *EGFR* mutations (3). A survey conducted by Robichaux et al. (2021) identified L747P as a PACC-class (P-loop and αC-helix compressing) variant, which was thought to be more effective against afatinib than any other TKI classes (15). Combined with the efficacy data of our case in the real world, we suggest that *EGFR* E746_L747delinsIP may be sensitive to afatinib, which is comparable to L747P.

In 2020, a retrospective study found that the NSCLC patients with uncommon *EGFR* exon 19 delins had significantly longer mPFS than those with the common exon 19 del with EGFR-TKI treatment (16). The patients in that study all presented *EGFR* exon19 in-frame deletion, which is unlike the complex variant in our case that did not cause any changes in amino acid length (**Supplementary 1**). *EGFR* exon 19 del at the K745-I759 position increases the kinase activity of EGFR, leading to the downstream pro-survival pathway hyperactivity, and consequently confers *EGFR* oncogenicity, which is sensitive to EGFR-TKIs (17, 18). Thus, the sensitivity of *EGFR* exon 19 rare complex delins mutation, in which the amino acid effect is missense mutations, to EGFR-TKIs warrants further investigation.

Our study had some limitations. First, the first-generation EGFR-TKI resistance mechanism of the *EGFR* E746_L747delinsIP should be supported by additional clinical data and functional studies. Second, large-scale data are required to support the use of afatinib to treat LUAD with *EGFR* E746_L747delinsIP, and a longer follow-up is needed to track the effect of afatinib on *EGFR* E746_L747delinsIP.

In conclusion, our case firstly showed that patients with rare *EGFR* E746_L747delinsIP mutated NSCLC-peritoneal metastases may be sensitive to afatinib and resistant to first-generation gefitinib and icotinib therapies. We suggest that first-generation EGFR-TKIs should be cautiously applied to patients with this *EGFR* exon 19 mutation, which causes amino acid substitution. Clinical trials are needed to develop treatment strategies for NSCLC harboring *EGFR* E746_L747delinsIP, and prospective or clinical studies are required to support these preliminary findings.

## Data Availability Statement

The datasets for this article are not publicly available due to concerns regarding participant/patient anonymity. Requests to access the datasets should be directed to the corresponding author.

## Ethics Statement

Ethical review and approval was not required for the study on human participants in accordance with the local legislation and institutional requirements. The patients/participants provided their written informed consent to participate in this study. Written informed consent was obtained from the individual(s) for the publication of any potentially identifiable images or data included in this article.

## Author Contributions

FK and DW: Conceptualization, Methodology, and Review. LY, LZ, and BS: Data collection and analysis, Writing, and Editing. YD and JY: Literature research. All authors contributed to the article and approved the submitted version.

## Funding

This work is supported by the National Natural Science Foundation of China (No. 81403220) and Tianjin Health and Family Planning-High Level Talent Selection and Training Project, National Key Research and Development (R&D) Plan (2018YFC1707400).

## Conflict of Interest

Authors LY, YD, JY, and DW were employed by YuceBio Technology Co., Ltd.

The remaining authors declare that the research was conducted in the absence of any commercial or financial relationships that could be construed as a potential conflict of interest.

## Publisher’s Note

All claims expressed in this article are solely those of the authors and do not necessarily represent those of their affiliated organizations, or those of the publisher, the editors and the reviewers. Any product that may be evaluated in this article, or claim that may be made by its manufacturer, is not guaranteed or endorsed by the publisher.

## Supplementary Material

Supplementary 1 Epidermal growth factor receptor tyrosine kinase inhibitor (EGFR-TKI) efficacy in non-small cell lung cancer (NSCLC) with rare EGFR exon 19 mutation.
