# Supplementary figures and images for: Case Report: Afatinib Sensitivity in Rare EGFR E746_L747delinsIP Mutated LUAD With Peritoneal Metastases
Source: Front Oncol. 2022 May 31;12:861271. doi: 10.3389/fonc.2022.861271 (PMC9194509; doi:10.3389/fonc.2022.861271)

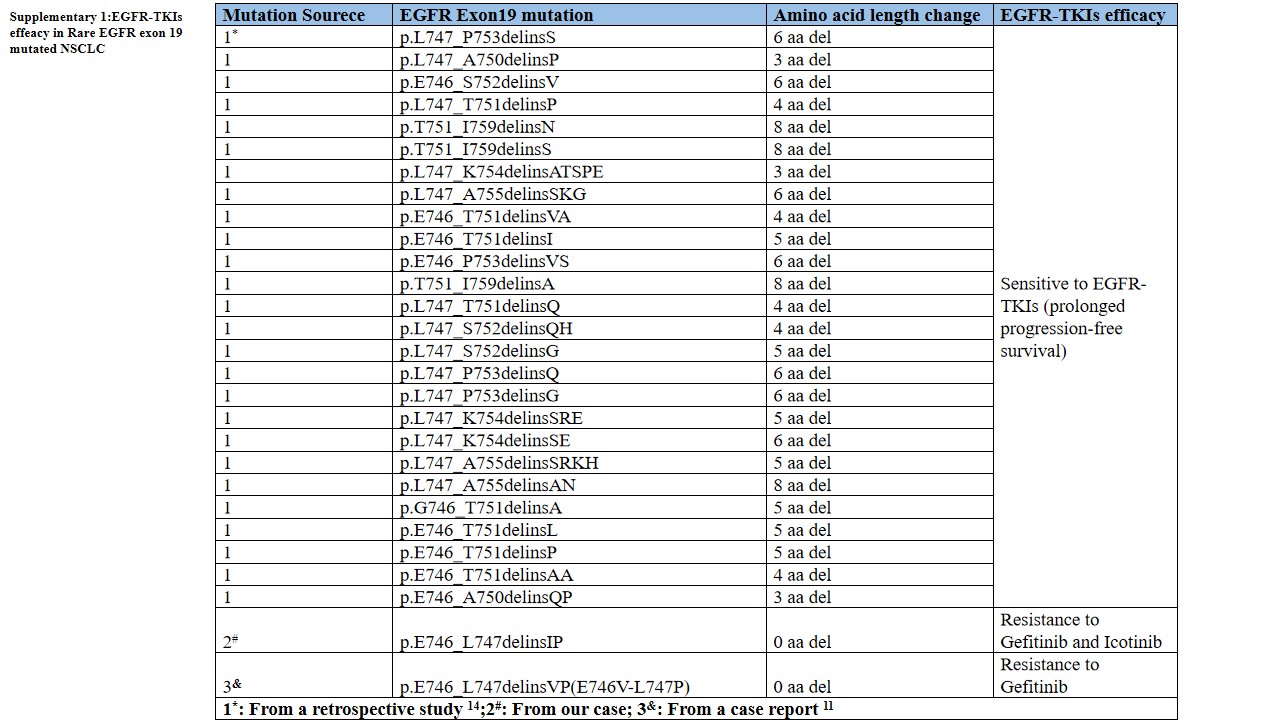

Supplement: Supplementary 1 — Epidermal growth factor receptor tyrosine kinase inhibitor (EGFR-TKI) efficacy in non-small cell lung cancer (NSCLC) with rare EGFR exon 19 mutation. [file Image_1.jpeg]
